# Influence of heart rate and heart rate variability on the feasibility of ultra-fast, high-pitch coronary photon-counting computed tomography angiography

**DOI:** 10.1007/s10554-023-02808-y

**Published:** 2023-02-11

**Authors:** Lukas T. Rotkopf, Matthias F. Froelich, Philipp Riffel, Christian H. Ziener, Carissa Reid, Heinz-Peter Schlemmer, Stefan O. Schoenberg, Isabelle Ayx

**Affiliations:** 1 Department of Radiology, German Cancer Research Center, Im Neuenheimer Feld 280, 69120 Heidelberg, Germany; 2 Medical Faculty, Ruprecht-Karls-University Heidelberg, Im Neuenheimer Feld 672, 69120 Heidelberg, Germany; 3 Department of Radiology and Nuclear Medicine, University Medical Center Mannheim, Heidelberg University, Theodor-Kutzer-Ufer 1-3, 68167 Mannheim, Germany; 4 Division of Biostatistics, German Cancer Research Center (DKFZ), Im Neuenheimer Feld 280, 69120 Heidelberg, Germany

**Keywords:** Photon-counting detector CT, Heart rate, FLASH mode, Dose reduction, Image quality

## Abstract

Coronary computed tomography angiography has become a mainstay in diagnosing coronary artery disease and is increasingly used in screening symptomatic patients. Recently, photon-counting computed tomography (PCCT) has been introduced into clinical practice, offering higher spatial and temporal resolution. As the applied radiation dose is highly dependent on the choice of scan mode and is lowest using the ultra-fast high-pitch (FLASH) mode, guidelines for their application are needed. From a retrospective study investigating the properties of a novel photon-counting computed tomography, all patients who underwent FLASH-mode PCCT angiography were selected between January and April 2022. This resulted in a study population of 46 men and 27 women. We recorded pre- and intrascan ECG readings and calculated heart rate (maximum heart rate 73 bpm) as well heart rate variability (maximum HRV 37 bpm) as measured by the standard deviation of the heart rate. Diagnostic quality and motion artifacts scores were recorded for each coronary artery segment by consensus between two readers. We found a highly significant association between heart rate variability and image quality (p < 0.001). The heart rate itself was not independently associated with image quality. Both heart rate and heart rate variability were significantly associated with the presence of motion artifacts in a combined model. Scan heart rate variability—but not heart rate itself—is a highly significant predictor of reduced image quality on high-pitch coronary photon-counting computed tomography angiography. This may be due to better scanner architecture and an increased temporal resolution compared to conventional energy-integrating detector computed tomography, which has to be addressed in a comparison study in the future.

## Introduction

Cardiac computed tomography has become a mainstay in the noninvasive diagnosis and assessment of coronary heart disease. Coronary computed tomography angiography (CCTA) is recommended for initial testing of patients suspected of having obstructive coronary artery disease (CAD) and may reduce the need for invasive coronary angiography (ICA) [1]. Adding CCTA to routine testing has been shown to lower the rate of a combined endpoint of cardiovascular death or myocardial infarction [2]. Initial CCTA instead of ICA has a similar risk of major adverse cardiovascular events, but a lower rate of major procedure-related complications [3]. However, CCTA is not recommended for patients with irregular heart rates or other conditions that may lead to lower image quality [1].

CCTA can be performed using different scan modes, with mode choice depending on heart rate and heart rate variability. Premedication with nitrates or beta-blockers can improve image quality significantly [4]. The lowest radiation dose is generally obtained by using the high-pitch mode, which has been made possible by the introduction of dual-source CT scanners and termed FLASH mode [5, 6]. By performing a single ECG-synchronized high-pitch scan of the whole heart radiation exposure is kept minimal. FLASH-mode CCTA can be performed if three requirements are met: the patient has both a low heart rate and a regular heart rhythm, as well as a high temporal resolution of the CT scanner. If these requirements are not met, the sequential scan mode is chosen, which demonstrates higher signal-to-noise ratios (SNR) but much higher dose exposures [7].

That thresholds for heart rate or heart rate variability above FLASH-mode scanning become inadvisable has been the subject of considerable research interest, with a broad range of found values [8–12]. A possible explanation are differences in scanner design and scanning parameters, as even third-generation CT scanners demonstrated higher SNR in FLASH mode than second-generation devices.

Recently, photon-counting detector computed tomography (PCCT) scanners have been introduced into clinical practice. The differences in detector design and resulting advantages over conventional energy-integrating detectors (EID) have been described in detail elsewhere [13, 14]. They can briefly be summarized as photon-counting detectors (PCD) having a higher inherent spatial resolution and photon efficiency, which can be used either to increase image quality or reduce radiation dose.

It is, therefore, necessary to reevaluate under which conditions FLASH-mode CCTA can be performed using PCCT scanners and how to optimize image quality and dose exposure. Herein, we investigated the influence of heart rate and heart rate variability on image quality and motion artifacts scores of FLASH-mode CCTA.

## Methods

### Study design

For this retrospective, single-center study, we included all patients who underwent, according to European Society of Cardiology (ESC) guidelines [1], clinically indicated cardiac computed tomography using a novel photon-counting computed tomograph between January and April 2022, who gave informed consent to be included in this study and for which the ultra-fast high-pitch mode (FLASH) was chosen. Patients were excluded in case of metal artifacts in the scan volume (n = 1) or in cases of active or former cardiac malignancies (n = 1). All investigations were conducted as part of a larger, IRB-approved study (ID 2021–659) investigating the properties of the novel PCCT scanner over a variety of use cases and were conducted according to the Declaration of Helsinki.

### Computed tomography imaging

All patients were scanned on a novel, first-generation dual-source photon-counting computed tomography scanner (Naeotom Alpha, Siemens Healthineers, Forchheim, Germany). All scans were performed using the electrocardiographic (ECG)-gated ultra-fast high-pitch (FLASH) mode with an effective rotation time of 0.25 s, a tube voltage of 120 kV, and automatic dose modulation. The choice of scan mode was left to the performing physician, guided by international guidelines and previous studies using conventional EID-CT scanners [8, 9]. If no contraindications were present, patients received 5–10 mg of intravenous metoprolol and 0.4—0.8 mg of sublingual nitroglycerine before the scan. First, a non-contrast-enhanced low-dose cardiac CT was performed for coronary calcium quantification. This scan was followed, independent of the result of the calcium quantification, by a contrast-enhanced CT angiography of the coronary arteries. For this, 80 ml of iodinated contrast agent (Imeron 400, Bracco Imaging Deutschland GmbH, Konstanz, Germany) were injected at a flow rate of 4—5 ml / s via an antecubital venous catheter followed by a 20 ml saline chaser (0.9% NaCl). Images were reconstructed using a vascular Bv40 kernel, a matrix size of 512 × 512, a variable FOV covering the whole heart and coronary vessels, a slice thickness of 0.6 mm, and a slice increment of 0.4 mm.

### Heart rate and heart rhythm assessment

Using the ECG readings provided by the scanner, heart rate was determined by the value directly during the scan. The heart rate variability was determined by the standard deviation of the five heartbeats prior to the scan.

### Image quality and motion artifact scores

**Fig. 1:**
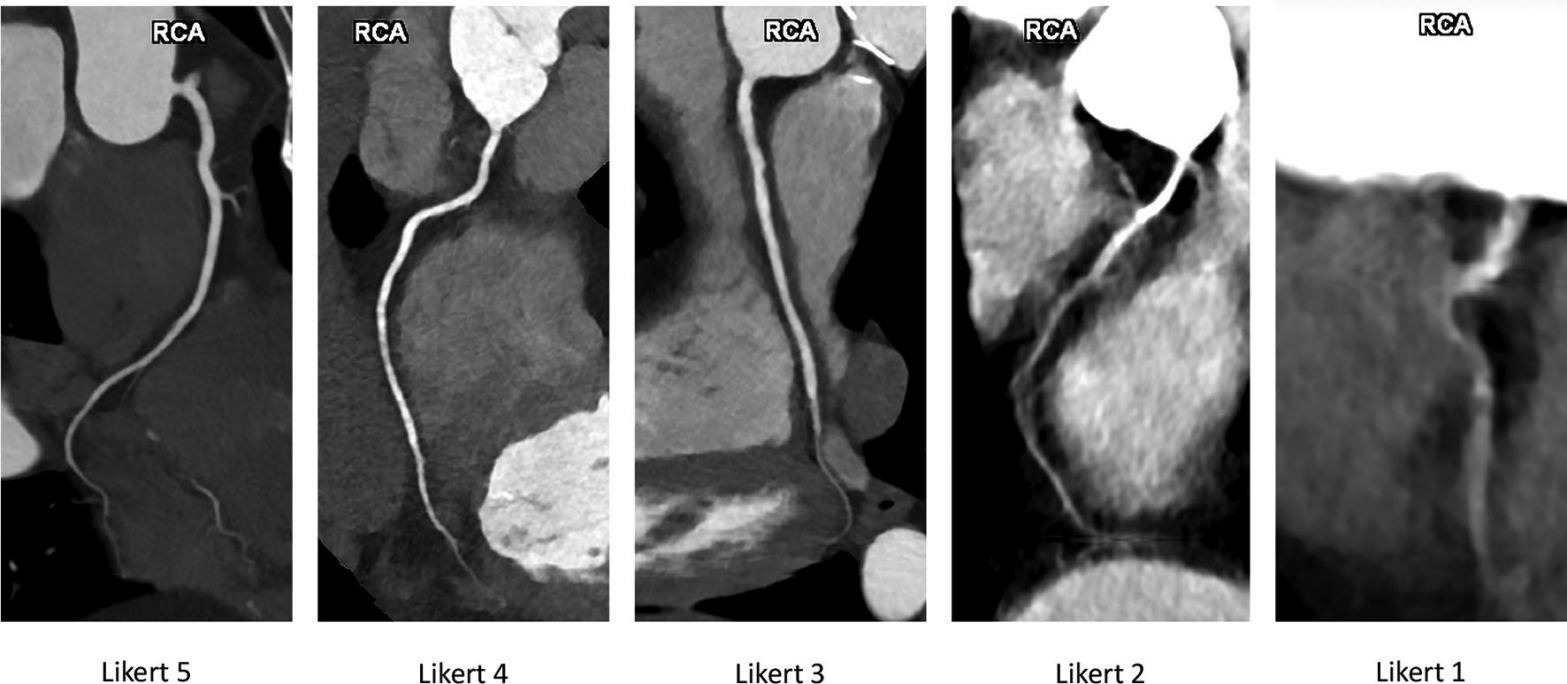
Illustration of diagnostic quality scores of the right coronary artery (RCA) on radial reconstructions of coronary photon-counting computed tomography angiography

Coronary arteries were divided into ten segments: left main (LM) coronary artery, proximal (pLAD), middle (mLAD), and distal left anterior descending (dLAD) coronary artery, proximal (pCX), middle (mCX), and distal circumflex (dCX) coronary artery, as well as proximal (pRCX), middle (mRCX), and distal right (dRCX) coronary artery. For each scan and each segment, both a diagnostic quality score (DQS) and a motion artifact score (MAS) were collected. The diagnostic quality score was graded according to the clinical adequacy of the coronary visualization. Scores of five to three corresponded to excellent, good, and moderate image quality with excellent, good and sufficient image quality, respectively. Scores of two corresponded to nondiagnostic quality with a visible coronary segment, and scores of one to nondiagnostic quality with poorly visible segments. An exemplary visualization of the DQS scores can be found in Fig. 1.

Motion artifact scores of zero corresponded to no motion artifacts, one to low to moderate and two to severe artifacts. Scores were determined as consensus between two radiologists (one consultant with nine years and one resident with three years of experience). The detailed score categories can be found in Table 1.

**Table 1:** Detailed score definition descriptions

Diagnostic quality score	
1	Non diagnostic image quality, segment is poorly visible
2	Non diagnostic image quality, segment is visible
3	Moderate image quality with sufficient intraluminal visibility
4	Good image quality with good intraluminal visibility
5	Excellent image quality with excellent intraluminal visibility
Motion artifact score	
0	No motion artifacts
1	Low to moderate motion artifacts
2	Severe motion artifacts

### Statistics

Summary statistics are reported as mean ± standard deviation for numerical and category percentages of categorical variables. Pearsons correlation coefficients were used to quantify the linear correlation between two variables. Linear mixed-effects models were used to control for patient identity and for vessel segment to account for making multiple measurements per scan. We calculated the Bayesian Information Criterion (BIC) and the log-likelihood as a model quality parameters.

All statistics were performed using R 4.2.1 (R Foundation, Vienna, Austria).

## Results

### Demographics

We included 73 patients (27 women), mean age 57 ± 12 years at exam, based on the inclusion criteria. The mean computed tomography dose index (CTDIvol) was 3.0 ± 1.1 mGy and the mean dose-length product (DLP) 56.5 ± 24.8 mGy cm for the coronary PCCT angiography scan. Further patient demographics and summary statistics can be found in Table 2.

**Table 2:** Study population and score distributions

Study population		
Number	73	–
Age	57 ± 12	Years
Sex (w)	27 (37%)	–
Scan parameters		
CTDIvol	3.0 ± 1.1	mGy
DLP	56.5 ± 24.8	mGy cm
Coronary CTA		
HR	58 ± 7	bpm
HRV	3.57 ± 5.50	bpm
Diagnostic quality score		
1	40 [5.6%]	–
2	107 [14.9%]	–
3	186 [25.9%]	–
4	240 [33.4%]	–
5	146 [20.3%]	–
Motion artifact score		
0	423 [58.9%]	–
1	207 [28.8%]	–
2	88 [12.3%]	–

*CTA* computed tomograpy angiography, *CTDIvol* computed tomography dose index, *DLP* dose-length product, *HR* heart rate, *HRV* heart rate variability

### Parameter and score distributions

Mean HR at CTA was 58 ± 7 beats per minute (bpm) with a range of 39–73 bpm. 15.0% of patients had an HR below or equal to 50 bpm and 5.4% above or equal to 70 bpm. Mean HRV was 3.5 ± 6.0 bpm with a range of 0.4–37 bpm.

For a total number of 719 vessels a DQS and for 718 vessels a MAS could be obtained. 40 (5.6%) vessels had a DQS of one, 107 (14.9%) of two, 186 (25.9%) of three, 240 (33.4%) of four, and 146 (20.3%) of five. 423 (58.9%) vessels had a MAS of zero, 207 (28.8%) of one, and 88 (12.3%) of two. Detailed parameter distribution per vessel can be found in Table 3.

**Table 3:** Diagnostic quality and motion artifact scores per vessel

	pRCA	mRCA	dRCA	LM	pLAD	mLAD	dLAD	pCX	mCX	dCX
Diagnostic quality score										
1	2 (2.78%)	3 (4.17%)	9 (12.50%)	0 (0%)	0 (0%)	0 (0%)	8 (11.11%)	0 (0%)	3 (4.17%)	15 (20.83%)
2	3 (4.17%)	7 (9.72%)	7 (9.72%)	0 (0%)	1 (1.39%)	8 (11.11%)	40 (55.56%)	2 (2.78%)	10 (13.89%)	29 (40.28%)
3	8 (11.11%)	29 (40.28%)	11 (15.28%)	3 (4.23%)	4 (5.56%)	40 (55.56%)	17 (23.61%)	18 (25.00%)	38 (52.78%)	18 (25.00%)
4	31 (43.06%)	20 (27.78%)	29 (40.28%)	26 (36.62%)	43 (59.72%)	23 (31.94%)	6 (8.33%)	36 (50.00%)	18 (25.00%)	8 (11.11%)
5	28 (38.89%)	13 (18.06%)	16 (22.22%)	42 (59.15%)	24 (33.33%)	1 (1.39%)	1 (1.39%)	16 (22.22%)	3 (4.17%)	2 (2.78%)
Motion artifact score										
0	47 (65.28%)	22 (30.56%)	46 (63.89%)	62 (87.32%)	59 (81.94%)	39 (54.17%)	31 (43.06%)	52 (72.22%)	35 (48.61%)	30 (42.25%)
1	12 (16.67%)	29 (40.28%)	12 (16.67%)	8 (11.27%)	11 (15.28%)	26 (36.11%)	34 (47.22%)	16 (22.22%)	25 (34.72%)	34 (47.89%)
2	13 (18.06%)	21 (29.17%)	14 (19.44%)	1 (1.41%)	2 (2.78%)	7 (9.72%)	7 (9.72%)	4 (5.56%)	12 (16.67%)	7 (9.86%)

*pRCA* proximal right coronary artery, *mRCA* middle right coronary artery, *dRCA* distal right coronary artery, *LM* left main coronary artery, *pLAD* proximal left anterior descending coronary artery, *mLAD* middle left anterior descending coronary artery, *dLAD* distal left anterior descending coronary artery, *pCX* proximal circumflex coronary artery, *mCX* middle circumflex coronary artery, *dCX* distal circumflex coronary artery

There were significant correlations between HR and HRV and between DQS and MAS (both p < 0.05), as can be seen in Table 4

**Table 4:** Correlations between variables and p-values for association significance

	HR	HRV	MAS	DQS
Correlations between variables				
HR	1.00			
HRV	− 0.36	1.00		
MAS	0.10	0.28	1.00	
DQS	− 0.02	− 0.18	− 0.55	1.00
Holms-corrected two-sided pairwise p-values				
HR	–			
HRV	< 0.001	–		
MAS	0.0086	< 0.001	–	
DQS	0.57	< 0.001	< 0.001	−

*HR* heart rate; HRV, heart rate variability; MAS, motion artifact score; DQS, diagnostic quality score

### Diagnostic quality score

**Fig. 2:**
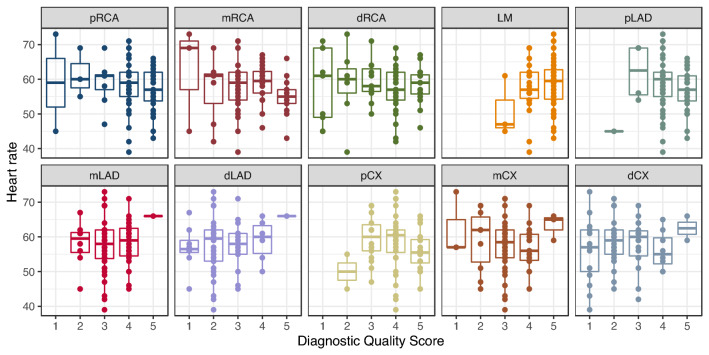
Heart rate depending on the diagnostic quality score broken down per vessel segment

**Fig. 3:**
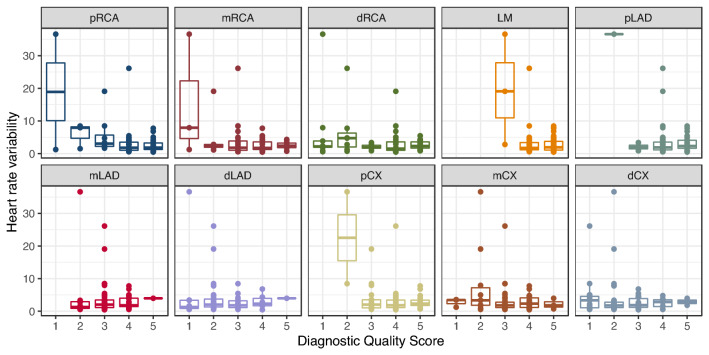
Heart rate variability depending on the diagnostic quality score broken down per vessel segment

The distribution of the DQS over the segments depending on the HR is shown in Fig. 2 and on the HRV in Fig. 3.

In linear mixed-effects models containing only each variable, HRV (model 2), but not HR (model 1) was a significant predictor of DQS (p < 0.001). In a combined mixed-effects model with both predictors (model 3), again only HRV was a significant predictor (p < 0.001). Model 2 had the lowest BIC, indicating that adding HR does not improve prediction accuracy. It can be seen from Fig. 3 that the association hinges mostly on outliers with high HRV. Detailed model parameters can be found in Table 5.

**Table 5:** Linear mixed-effects models for the diagnostic quality score controlled for vessel segment and patient identity

	Model 1		Model 2		Model 3	
	Coefficients	p-Values	Coefficients	p-Values	Coefficients	p-Values
HR	− 0.0028	0.76	–	–	− 0.016	0.087
HRV	–	–	− 0.039	0.00090	− 0.046	0.00021
BIC	1771.2	–	1760.0	–	1763.6	–
logLikelihood	− 869.1	–	− 863.5	–	− 862.0	–

*HR* heart rate, *HRV* heart rate variability, *BIC* Bayes information criterion

### Motion artifact score

**Fig. 4:**
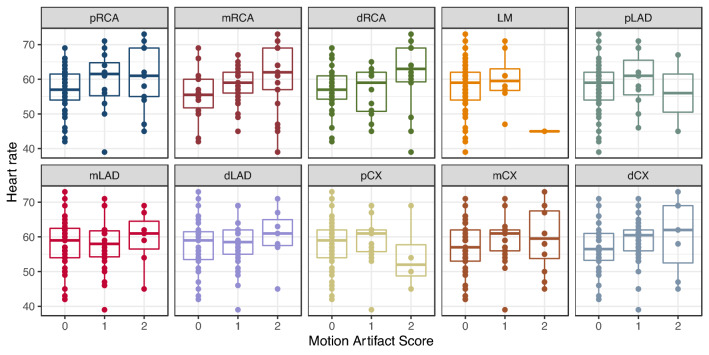
Heart rate depending on the motion artifact score broken down per vessel segment

**Fig. 5:**
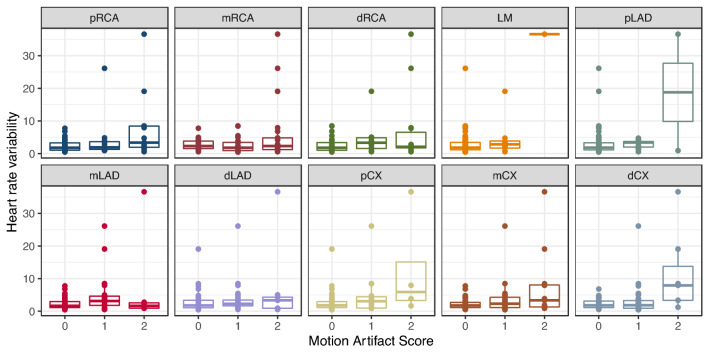
Heart rate variability depending on the motion artifact score broken down per vessel segment

The distribution of the MAS over the segments depending on the HR is shown in Fig. 4 and on the HRV in Fig. 5.

In linear mixed-effects models containing only each variable, only HRV (model 2), but not HR (model 1) was a significant predictor of the MAS (p < 0.001). In a combined mixed effects model (model 3), both predictors were significant (both p < 0.001). The combined model had a lower BIC than the other models, indicating that taking both into account improves prediction of the MAS compared to univariate models. Detailed model parameters can be found in Table 6.

**Table 6:** Linear mixed-effects models for the motion artifact score controlled for vessel segment and patient identity

	Model 1	Model 2	Model 3			
	Fixed effects	p-values	Fixed effects	p-values	Fixed effects	p-values
HR	0.010	0.13	–	–	0.024	0.00017
HRV	–	–	0.036	2.03e−5	0.048	4.8e−8
BIC	1340.8	1324.4	1316.4			
logLikelihood	− 653.9	− 645.7	− 638.4			

	Model 1		Model 2		Model 3	
	Coefficients	p-Values	Coefficients	p-Values	Coefficients	p-Values
HR	0.010	0.13	–	–	0.024	0.00017
HRV	–	–	0.036	2.03e−5	0.048	4.8e−8
BIC	1340.8	1324.4	1316.4			
logLikelihood	− 653.9	− 645.7	− 638.4			

*HR* heart rate, *HRV* heart rate variability, *BIC* Bayes information criterion

## Discussion

In this study, we investigated the influence of HR and HRV on diagnostic quality and presence of motion artifacts in cardiac photon-counting computed tomography angiography. We found no evidence of a significant association of HR with image quality scores (p > 0.05) but observed a strong influence of the HRV (p < 0.001) in a combined mixed-effects model. Both HR and HRV were significantly associated with the presence of motion artifacts (both p < 0.001) in a combined model.

We chose to define heart rate variability in terms of the standard deviation of the five heartbeats preceding the acquisition of the CT angiography. This is equivalent to the commonly applied standard deviation of the normal R-R intervals (SDNN). We obtained a mean SDNN of 55.2 ± 68.1 ms, which is in line with previous studies in patients with compromised cardiac health [15, 16].

The influence of HRV on both diagnostic quality and motion artifact scores is plausible from a mechanistic point of view. Variations in the R-R intervals prior to the scan lead to mistiming of the acquisition, which then falls, instead of the ideal mid-diastole, in intervals of the heart cycle with increased cardiac wall movement. Elevated HR itself does not inherently lead to mistimed acquisitions but shortens the ideal time interval. Accordingly, while the HR is a significant predictor of the motion artifact score, the correlation coefficient is very low (Pearson’s correlation coefficient 0.10). Furthermore, due to this weak association, HR does not become a predictor of reduced image quality, despite the significant correlation between MAS and DQS.

The lack of an association between image quality scores and HR may also be due to the high temporal resolution of the CT scanner architecture. This is supported by a study showing that FLASH-mode image quality increased significantly between second- and third-generation dual-source CT scanners [17]. These improvements may have increased the threshold above which HR starts impacting diagnostic quality above the highest HR included in our study. It is also possible that properties of the PCD itself may lead to better image quality at a given HR. At the same dose, the higher photon efficiency leads to lower image noise [13], possibly shifting the threshold above which unacceptable decreases in image quality occur. Furthermore, the generated virtual monoenergetic images improve iodine contrast, which may allow better differentiation between cardiac vessels and tissue [18]. Only 5.4% of the included patients had a HR of 70 or more, and the highest recorded heart rate at the scan was 73. Therefore, further studies including patients with higher HR are indicated.

In the literature, varying results for associations between image quality and HR or HRV have been found. A recent study demonstrated FLASH-mode scanning is possible for the majority of pediatric patients with heart rates < 100 bpm with adequate proximal coronary visualization [8]. In another study, average HR and HRV were significantly higher in patients with at least one nondiagnostic segment, and all patients with HR < 64 bpm and HRV < 13 bpm, as defined by the difference between the maximum and minimum HR, had diagnostic image quality in all segments [10]. Another study found that HR and motion artifacts on the preceding coronary artery calcium quantification scan are independent predictors of image quality [11]. In a combined phantom-patient study, distortion of coronary arteries was less than 1 mm for HR up to 75 bpm in the phantom and the depiction of patient coronary segments was of diagnostic image quality for all patients with HR up to 73 bpm [9]. The latter study, in which the phantom part was performed using a third-generation dual-source CT system (Somatom Force, Siemens Healthineers. Forchheim, Germany), is most in line with our study. Especially their results of the influence of the simulated HR on the distortion vector of the coronary arteries (see Fig. 3 there) show almost no influence up to 75 bpm, after which the magnitude increases. Given that the range of HR seen in our study is below this threshold, it should come as no surprise that no significant association with HR was observed.

Our study has several limitations. The main limiting factor is the retrospective nature and the restriction on the analysis of patients who received only FLASH scans. As the choice of scan mode was left to the performing physician, patients with significant arrhythmia or elevated heart rates were naturally excluded, leading to significant selection bias. However, as no fixed threshold was set beforehand, borderline cases were indeed included, as can be seen in the relatively high number of patients with HR for which normally a different scan mode would be chosen. Additionally, possible changes in HR and HRV between the timepoint of mode selection and the scan would inherently fuzzy such a threshold. Another factor is the inherent subjectivity of image quality and motion artifact scoring. While the scores were determined in consensus, a study with multiple readers would possibly reduce reading bias. Minor limiting factors were the relatively low patient number, possible temporal changes in heart rhythm due to the different amount of administered premedication, and the inclusion of patients both with and without known coronary, structural or functional heart disease. Furthermore, we did not perform a direct comparison between cardiac CTs performed on either EID-CT or PCCT scanners, which would provide further information on possible differences in the HR threshold.

This preliminary study represents the first investigation regarding the possible selection of scan mode in photon-counting cardiac CT imaging. Due to the retrospective and preliminary nature, changes in mode selection or threshold values can and should not be drawn. Further investigations with possible randomization or comparison between scan modes and inclusion of patients with higher heart rates are urgently necessary to improve the strength of evidence and provide clinical guidelines.

## Conclusion

We could demonstrate that the scan heart rate variability, but not the mean heart rate during FLASH-mode PCCT coronary angiography is significantly associated with reduced subjective diagnostic quality and increased motion artifacts in a preliminary study with a maximum HR of 73 bpm and maximum HRV of 37 bpm. This has implications for the selection of scan mode choice and subsequently possibly reductions in mean applied radiation dose. The results are in line with previous results investigating last-generation conventional energy-integrating CT, and it is possible that PCCT scanners may push the HR threshold above which motion artifacts begin influencing image quality even higher.

## Abbreviations

AIC: Akaike Information Criterion
BIC: Bayesian Information Criterion
bpm: Beats per minute
CHD: Coronary heart disease
CT: Computed tomography
CTDIvol: Computed tomography dose index
CVD: Cardiovascular disease
DICOM: Digital Imaging and Communications in Medicine
DLP: Dose-length product
DQS: Diagnostic quality score
ECG: Echocardiography
EID: Energy-integrating detector
ESC: European Society of Cardiology
HR: Heart rate
HR-SD: Standard deviation of the heart rate
MAS: Motion artifact score
PCCT: Photon-counting computed tomography
PCD: Photon-counting detector
SD: Standard deviation
SNR: Signal-to-noise ratio
